# Clinical characterization of adolescent girls linked to puberty menorrhagia

**DOI:** 10.6026/973206300220571

**Published:** 2026-01-31

**Authors:** Rowson Ara, Salma Akter Munmun, Shahinoor A.M, Sabiha Islam, Farah Noor, Reefaat Rahman, Sayada Fatema Khatun

**Affiliations:** 1 Department of Obstetrics and Gynecology, Bangladesh Medical University (BMU), Dhaka, Bangladesh; 2 Department of Gynecological Oncology, Bangladesh Medical University (BMU), Dhaka, Bangladesh; 3 Department of Pediatric Surgery, Bangladesh Medical University (BMU), Dhaka, Bangladesh

**Keywords:** Puberty menorrhagia, adolescent gynecology, anovulatory DUB, hormonal therapy, anemia

## Abstract

Clinical characterization of adolescent girls linked to puberty menorrhagia is relevant. Hence, a hospital-based observational study was conducted on 76 adolescent girls diagnosed with puberty menorrhagia. Patients underwent detailed clinical assessments, laboratory investigations and imaging studies to determine the underlying causes. The most common cause of puberty menorrhagia was anovulatory DUB (72.37%), followed by polycystic ovarian disease (10.53%), hypothyroidism (6.58%) and hematological disorders (6.58%). A significant proportion of patients experienced prolonged bleeding (7-8 days in 72.37% of cases), with moderate-to-severe anemia affecting nearly two- thirds of the cohort. Thus, it is important to clinically characterize adolescent girls presenting with puberty menorrhagia.

## Background:

Puberty menorrhagia is a significant clinical concern in adolescent girls, characterized by excessive menstrual bleeding occurring during puberty [1]. This condition affects a substantial proportion of adolescent females globally, with an estimated prevalence ranging from 14% to 48%, impacting both physical health and quality of life [2]. Heavy menstrual bleeding at this stage can lead to anemia, fatigue and psychological distress, affecting academic performance and daily activities [3]. While menstrual irregularities are common in adolescence due to the immaturity of the hypothalamic-pituitary-ovarian (HPO) axis, persistent or excessive bleeding requires medical attention [2]. The physiological process of menarche, marking the onset of menstruation, occurs as a result of hormonal regulation involving the hypothalamus, pituitary gland and ovaries [4]. However, during the initial years after menarche, the hormonal axis remains immature, often leading to anovulatory cycles and irregular bleeding patterns [5]. Puberty menorrhagia occurs when menstrual bleeding exceeds 80 mL per cycle or lasts more than seven days, significantly deviating from normal physiological variations [6]. In most cases, the underlying cause is dysfunctional uterine bleeding (DUB), which results from anovulation and unopposed estrogen stimulation leading to excessive endometrial proliferation and subsequent irregular shedding [7]. Endocrine dysfunctions, including thyroid disorders and polycystic ovarian syndrome (PCOS), can lead to menstrual irregularities and prolonged bleeding [8]. Moreover, infections, underlying systemic illnesses and the use of specific medications, such as anticoagulants or hormonal therapies, should be carefully considered in the differential diagnosis to ensure accurate identification and appropriate management of the condition [9]. Chronic blood loss can lead to iron deficiency anemia, causing pallor, fatigue, dizziness and reduced exercise tolerance, with severe cases requiring blood transfusions [10]. The psychological impact of heavy menstrual bleeding is also considerable, as it can lead to anxiety, embarrassment and absenteeism from school and social activities [11]. Timely diagnosis and intervention are essential to prevent complications, requiring a thorough clinical assessment, including menstrual history, family history of bleeding disorders and physical examination [12]. Laboratory investigations such as complete blood count, coagulation profile and hormonal assays help identify underlying hematologic or endocrine abnormalities [13]. In cases where structural causes are suspected, imaging modalities like pelvic ultrasound may be required to assess uterine and ovarian pathology [14]. Hormonal therapy, including combined oral contraceptives or progesterone-only regimens, is commonly used to regulate the menstrual cycle and reduce blood loss [5]. Iron supplementation is commonly required to treat anemia, helping to restore iron levels, improve red blood cell production and alleviate associated symptoms like fatigue and weakness [3]. In cases where coagulation disorders are identified, specific treatments such as desmopressin or clotting factor replacement therapy may be required [15]. Despite the clinical importance of puberty menorrhagia, there remains a lack of comprehensive data on its prevalence, etiological factors and management outcomes in many regions [16]. Therefore, it is of interest to report the underlying causes, clinical manifestations and therapeutic approaches observed among adolescent girls presenting with puberty menorrhagia.

## Materials and Methods:

This observational study was conducted at Department of Obstetrics and Gynecology, Bangladesh Medical University (BMU), Dhaka, Bangladesh to evaluate adolescent girls presenting with puberty menorrhagia. A total of 76 adolescent girls presenting with menorrhagia were selected to participate between January 2023 December 2024.

## Inclusion criteria:

[1] Girls aged between menarche and 19 years.

[2] Women visited the outpatient department or were admitted to the hospital with complaints of excessive menstrual bleeding.

[3] Blood loss was considered excessive if the menstrual cycle lasted more than seven days or if there was a history of clot passage.

## Exclusion criteria:

[1] Patients with incomplete or missing menstrual history.

[2] Patients with known cases of Bleeding diathesis, Hypothyroidism or hyperthyroidism, Tuberculosis (active or history of contact), Polycystic ovary syndrome (PCOS), Hypertension, Diabetes mellitus, Asthma, Other chronic medical illnesses, Bleeding disorders.

[3] Patients on medications affecting menstrual cycles.

[4] Patients with incomplete or missing laboratory investigations, including Complete blood count (CBC), Peripheral smear, Random blood sugar (RBS), Coagulation profile (PT/PTT/BT/CT/platelet count), Thyroid function tests (T3, T4, TSH), Prolactin level, LH/FSH level, Ultrasonography findings.

## Ethical consideration:

The study received approval from the Ethics Committee of Institutions. Participation was voluntary and confidentiality of participant information was strictly maintained. Prior to participation, the objectives of the study were explained to the girls and informed verbal consent was obtained.

## Data collection:

A detailed history was taken from each participant, covering various aspects essential for data collection. Demographic and growth information included age, BMI, socioeconomic status. Menstrual history was documented, noting the age of menarche, duration of menstrual cycles, volume of blood loss and number of pads used daily, number of days of bleeding. Etiology, Hb level and management procedure were also documented. Medical history was also obtained, including details on weight changes, voice changes, tuberculosis, endocrine disorders such as diabetes and thyroid dysfunction, medication use and any cardiac, renal, or hematological conditions. Additionally, records of previous blood transfusions or surgical interventions were documented.

## Physical examination:

Each participant underwent a thorough clinical evaluation to assess their overall health and identify any underlying conditions. The general examination included measurements of height, weight and BMI, along with the assessment of pallor, icterus, signs of malnutrition and vitamin deficiencies. A systemic examination was conducted to evaluate neck veins, glands, gum bleeding, pulse, blood pressure and temperature. The abdominal examination involved palpation for hepatosplenomegaly, ascites, or any abdominal-pelvic masses. Additionally, the skin and joint examination focused on detecting purpuric spots, bony tenderness and joint swelling. Hormonal assessment was performed by observing signs of hyperandrogenism, such as acne and hirsutism, as well as evaluating secondary sexual characteristics, including breast development and axillary or pubic hair. The gynecological examination involved an inspection of the vulva, while vaginal examinations were avoided for patients with an intact hymen.

## Laboratory and diagnostic investigations:

All participants underwent baseline laboratory tests to evaluate their health status and identify potential underlying causes of menorrhagia. Routine tests included a urine pregnancy test, complete blood count (CBC), hemoglobin (Hb) levels, platelet count, coagulation profile, blood grouping and Rh typing. Imaging studies such as transabdominal ultrasound (USG) and serial folliculometry were conducted to assess ovulation status. Additional tests, including blood sugar, thyroid profile and hormonal assays (LH, FSH and prolactin), were performed in selected cases. Patients suspected of tuberculosis underwent Mantoux testing and chest X-rays for further evaluation. Advanced investigations were carried out in specific cases, including menstrual blood PCR for Mycobacterium tuberculosis antigen, endometrial biopsy following dilatation and curettage, bone marrow examination, serum ferritin levels and hemoglobin electrophoresis. Specialized tests such as examination under anesthesia (EUA) and laparoscopy were considered for unmarried patients and those with inconclusive imaging findings. Furthermore, additional investigations like Von Willebrand factor activity, Ristocetin cofactor assay and 21-day serum progesterone level were conducted in suspected cases to determine the underlying pathology.

## Management approach:

The management approach was tailored to each patient based on their condition and the underlying cause of menorrhagia. Pharmacological treatment was the primary intervention; with hemodynamically stable patients experiencing anovulatory bleeding initially managed using antifibrinolytic drugs such as tranexamic acid during menstruation. In cases that did not respond to initial therapy, hormonal treatments, including combined oral contraceptive pills (COCs) or progesterone, were administered. Anemia was addressed depending on the severity of the condition. Additionally, supportive care was provided to all patients, emphasizing nutritional guidance, physical well- being and psychological support to improve overall health and quality of life.

## Follow-up and monitoring:

Regular follow-ups were scheduled to track menstrual patterns, clinical progress and treatment response. Patients maintained menstrual calendars and periodic evaluations ensured effective management and well-being.

## Data analysis:

Data analysis was conducted using SPSS software version 26.0 (SPSS Inc., Chicago, IL). Descriptive statistics were used to summarize the characteristics of the participants. Categorical variables were expressed as frequencies and percentages, while continuous variables were reported as means with standard deviations. Comparisons between groups were performed using Student's t-test. A p-value of <0.05 was considered statistically significant.

## Results:

**Figure 1:**
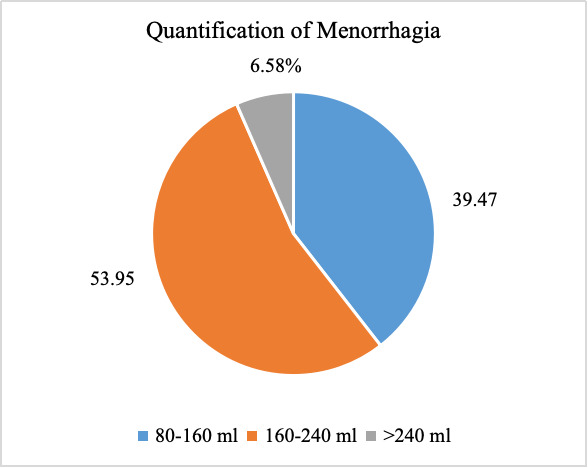
Quantification of menorrhagia among women (n=76)

**Figure 2:**
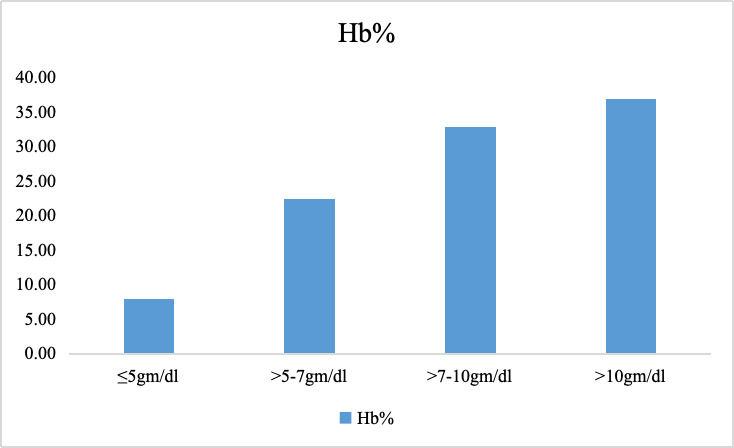
Hemoglobin levels in the study participants (n=76)

A total of 76 patients participated in this study. The majority of participants were between 17-19 years of age (42.11%), followed by those aged 14-16 years (34.21%) and 23.68% were under 14 years. Regarding BMI, most participants had a normal BMI (76.32%), while 14.47% were underweight and 9.21% were overweight. The majority of participants had their menarche between 12-13 years (42.11%), with 35.53% having menarche after 13 years (Table 1). The majority of participants had symptoms for more than 12 months (46.05%), with 34.21% experiencing symptoms for 6-12 months and 19.74% for less than 6 months. Regarding the number of pads used per day, most women used 5-6 pads (52.63%), followed by 3-4 pads (42.11%) and a few used more than 6 pads (5.26%). Most women reported bleeding for 7-8 days (72.37%), followed by those bleeding for 5-6 days (14.47%) and more than 8 days (13.16%) (Table 2). Figure 1 demonstrated that most women (53.95%) had blood loss in the range of 160-240 ml, while 39.47% had blood loss between 80-160 ml and 6.58% experienced blood loss greater than 240 ml. Table 3 presented the causes of the disease. Anovulatory dysfunctional uterine bleeding (DUB) was the most common cause, affecting 72.37% of the participants. Other causes included PCOD (10.53%), hypothyroidism (6.58%), ITP (2.63%), genital tuberculosis (2.63%), fibroids (2.63%), von Willebrand disease (1.32%) and cervical polyps (1.32%). A significant proportion (36.84%) had Hb levels greater than 10 gm/dl, with 32.89% between 7-10 gm/dl, 22.37% between 5-7 gm/dl and 7.89% with Hb levels ≤5 gm/dl (Figure 2). The most common hormonal treatment was COC (43.42%), followed by progesterones and COC (42.11%) and progesterones alone (14.47%). Hematinics were administered to half of the participants (50%), with tranexamic acid given to 35.53%. Smaller proportions received metformin (6.58%), thyroxine (3.95%) and blood transfusions (3.95%) (Table 4).

## Discussion:

The findings of this study provide valuable insights into the clinical characteristics, etiological factors and management strategies for adolescent girls with puberty menorrhagia in a tertiary-level hospital in Bangladesh. Regarding the age distribution, the majority of participants were between 17-19 years of age (42.11%), followed by those aged 14-16 years (34.21%) and 23.68% were under 14 years. Smith *et al.* in their study with 100 participants show a more evenly distributed age range, with only 10% falling within the 17-19 age group [17]. Another study reveals a higher percentage (50%) of participants under the age of 14 [18]. This distribution suggests that puberty menorrhagia is more commonly reported in older adolescents, potentially due to increasing awareness and healthcare-seeking behavior in this age group. The BMI distribution within our cohort shows that the majority fall within the normal BMI range. However, the presence of participants with a BMI exceeding 25 indicates a potential association between higher BMI and menorrhagia. Study indicated that obesity is associated with heavy menstruation [19]. The age of menarche also varied, with most participants (42.11%) experiencing menarche between 12-13 years and 35.53% after 13 years. Early menarche (<12 years) was relatively uncommon, reinforcing that the hormonal immaturity of the HPO axis during the initial post-menarche years contributes significantly to anovulatory cycles and resultant menorrhagia. Average age of menarche in an Asian country India is 12.5 years which is comparable to our study [20]. 34.21% of the patients in our study had symptoms for 6 months to 1 year and 46.05% had symptoms for more than 1 year which is comparable with other studies [21]. Regarding the number of pads used per day, 52.63% of participants required 5-6 pads daily, suggesting moderate-to-severe blood loss. The duration of menstrual bleeding was also evaluated, revealing that the majority (72.37%) experienced 7-8 days of bleeding, which exceeds the normal menstrual duration (4-7 days). Quantification of menorrhagia was 160-240ml for 53.95% patients in this study. The etiological analysis revealed that anovulatory DUB was the most prevalent cause (72.37%). This is similar to the observations made by Gillani S & Mohammad S. (74.28%) in our study PCOD comprises the second commonest cause of puberty menorrhagia which is similar to Gillani S & Mohammad S. (8.6%) [22]. Other studies have regarded bleeding diathesis as second common cause of puberty menorrhagia. Hemoglobin is a critical component of red blood cells responsible for transporting oxygen throughout the body. A decrease in hemoglobin levels may lead to anemia; a condition associated with fatigue, weakness and diminished overall health [23]. In the term of anemia severity, 36.84% of participants had hemoglobin levels greater than 10 gm/dL, while 32.89% had levels between 7-10 gm/dL, 22.37% between 5-7 gm/dL and 7.89% had severe anemia (Hb ≤5 gm/dL). The relatively high proportion of patients with moderate-to-severe anemia underscores the long-term impact of chronic blood loss. Treatment modalities varied among participants, with combined oral contraceptives (COCs) being the most commonly prescribed hormonal therapy (43.42%), followed by progesterone-only therapy (14.47%) and a sequential regimen of progesterone followed by COCs (42.11%). Hematinics were administered to 50% of patients to address anemia, while tranexamic acid was used in 35.53% of cases to reduce menstrual blood loss. A smaller subset of patients received metformin (6.58%), thyroxine (3.95%), or blood transfusions (3.95%), reflecting the need for individualized treatment approaches based on underlying pathology. Despite the effectiveness of pharmacological interventions, a small subset of patients (3.95%) required blood transfusions, reflecting the severity of anemia in some cases. This highlights the need for improved early detection and iron supplementation strategies to prevent severe hematologic consequences in this population. Treatment strategies are comparable with the study of Gillani & Mohammad (2012) [22]. There were some limitations in this study. The short duration makes it difficult to assess long-term outcomes, complications, or recurrence rates. Certain hematological and genetic factors contributing to menorrhagia were not extensively evaluated. Patient self-reporting of menstrual blood loss may introduce recall bias. Future investigations should prioritize multi-center studies to gain deeper insights into the pathophysiology of threatened abortion and to assess possible interventions aimed at enhancing both maternal and fetal outcomes.

## Conclusion:

We report the clinical characteristics, etiological factors and management approaches for puberty menorrhagia in adolescent girls, with anovulatory dysfunctional uterine bleeding (DUB) emerging as the most common cause. The findings emphasize the importance of early diagnosis and appropriate intervention, including hormonal therapy, antifibrinolytics and iron supplementation, to prevent complications such as anemia and impaired quality of life. While pharmacological management remains the primary treatment approach, a multidisciplinary strategy involving gynecologists, endocrinologists and hematologists is essential for optimal patient outcomes. Further large-scale, multi-center study is needed to enhance the understanding of puberty menorrhagia and refine evidence-based treatment protocols.

## Figures and Tables

**Table 1:** Demographic details of the study population (n=76)

**Variables**	**Frequency (n)**	**Percentage (%)**
**Age (years)**		
<14	18	23.68
14-16	26	34.21
17-19	32	42.11
**BMI**		
<18.5	11	14.47
18.5-25	58	76.32
>25	7	9.21
**Age of Menarche (years)**		
<10	1	1.32
11-Oct	3	3.95
12-Nov	13	17.11
13-Dec	32	42.11
>13	27	35.53

**Table 2:** Disease characteristics among women (n=76)

**Variables**	**Frequency (n)**	**Percentage (%)**
**Duration of symptoms (months)**		
<6	15	19.74
12-Jun	26	34.21
>12	35	46.05
**No. of pads used per day**		
4-Mar	32	42.11
6-May	40	52.63
>6	4	5.26
**No. of days of bleeding**		
6-May	11	14.47
8-Jul	55	72.37
>8	10	13.16

**Table 3:** Causes of disease among patients (n=76)

**Causes**	**Frequency (n)**	**Percentage (%)**
Anovulatory DUB	55	72.37
PCOD	8	10.53
Hypothyroidism	5	6.58
ITP	2	2.63
Genital TB	2	2.63
Cervical Polyp	1	1.32
Fibroid	2	2.63
Von Willebrand disease	1	1.32

**Table 4:** Treatment received by study subjects (n=76)

**Treatment**	**Frequency (n)**	**Percentage (%)**
**Hormones used**		
COC	33	43.42
Progesterones	11	14.47
Progesterones followed by COC	32	42.11
**Drug given along with hormonal treatment**		
Hematinics	38	50
Tranexamic acid	27	35.53
Metformin	5	6.58
Thyroxine	3	3.95
Blood transfusion	3	3.95
